# Thoracic endovascular aortic repair for complicated chronic type B aortic dissection in a patient on hemodialysis with recurrent ischemic colitis

**DOI:** 10.1186/s40792-016-0165-2

**Published:** 2016-04-18

**Authors:** Yuko Miyazaki, Tadashi Furuyama, Yutaka Matsubara, Keiji Yoshiya, Ryosuke Yoshiga, Kentaro Inoue, Daisuke Matsuda, Yukihiko Aoyagi, Masaaki Kato, Takuya Matsumoto, Yoshihiko Maehara

**Affiliations:** Department of Surgery and Science, Graduate School of Medical Sciences, Kyushu University, 3-1-1 Maidashi, Higashi-ku, Fukuoka, 812-8582 Fukuoka Japan; Department of Cardiovascular Surgery, Morinomiya Hospital, Osaka, Japan

**Keywords:** Thoracic endovascular aortic repair, Chronic type B aortic dissection, Ischemic colitis, Malperfusion, Surgical intervention

## Abstract

We present a successful case of thoracic endovascular aortic repair (TEVAR) for chronic Stanford type B aortic dissection (B-AD) with recurrent ischemic colitis. The patient was a 56-year-old woman with abdominal pain as the main complaint who had two operations previously: the total arch replacement 8 years ago and the Bentall 7 years ago for acute Stanford type A aortic dissection. Her abdominal pain worsened as her blood pressure became low during her hemodialysis treatment. An enhanced computed tomography scan was performed on the patient and showed chronic B-AD that occurred from the distal anastomotic part of the total arch graft to the bilateral common iliac arteries. The celiac artery and superior mesenteric artery (SMA) arose from the true lumen, and these were compressed by the expanded false lumen. Her complicated chronic B-AD was treated with the Zenith Dissection Endovascular System, and its procedure was performed as her proximal entry tear was covered by a proximal tapered Zenith TX2 stent graft, supplemented by a noncovered aortic stent extending across both renal arteries, the SMA, and the celiac artery. Seven days after this operation, enhanced computed tomography showed that the patient’s true lumen was expanded and her blood flow to the true lumen and SMA was improved. On the other hand, her false lumen tended to be thrombosed. Consequently, she was discharged 10 days after the operation without any postoperative complications as she had no abdominal complaints even though she underwent hemodialysis three times per week after the operation. We believe that TEVAR supplemented by a noncovered aortic stent is an effective treatment, even for highly chronic B-AD in dialysis patients.

## Background

Endovascular techniques and devices have recently been developed for the management of aortic dissection [1–3]. Recently, the INSTEAD-XL study showed that thoracic endovascular aortic repair (TEVAR) can improve the survival rate of up to 5 years for aorta-specific cases and delayed the disease progression of stable Stanford type B aortic dissection (B-AD) with suitable anatomy [4, 5] even though B-AD can be complicated by various symptoms such as uncontrollable hypertension, aortic rupture, and end-organ ischemia [4, 5]. However, the effectiveness of TEVAR for such complicated chronic B-AD of patients is still an ongoing debate. Additionally, patients with end-stage renal failure (ESRF) on hemodialysis have many atherosclerotic comorbidities including ischemic colitis [6].

We present here a rare case of chronic B-AD in a dialysis patient. The patient had been followed up for 8 years and developed ischemic colitis repeatedly along with hypotension due to hemodialysis. We performed TEVAR with a noncovered aortic stent for this complicated chronic B-AD.

## Case presentation

The patient was a 56-year-old woman with recurrent abdominal pain as the main complaint and who had been on maintenance hemodialysis of ESRF for 26 years. She had the operations of total arch replacement 8 years ago and a Bentall 7 years ago for acute Stanford type A aortic dissection. Although dissections still remained on the descending thoracic and abdominal aorta from the previous two operations, the diameter of the aorta remained stable without its enlargement. Therefore, she underwent conservative treatments.

**Fig. 1:**
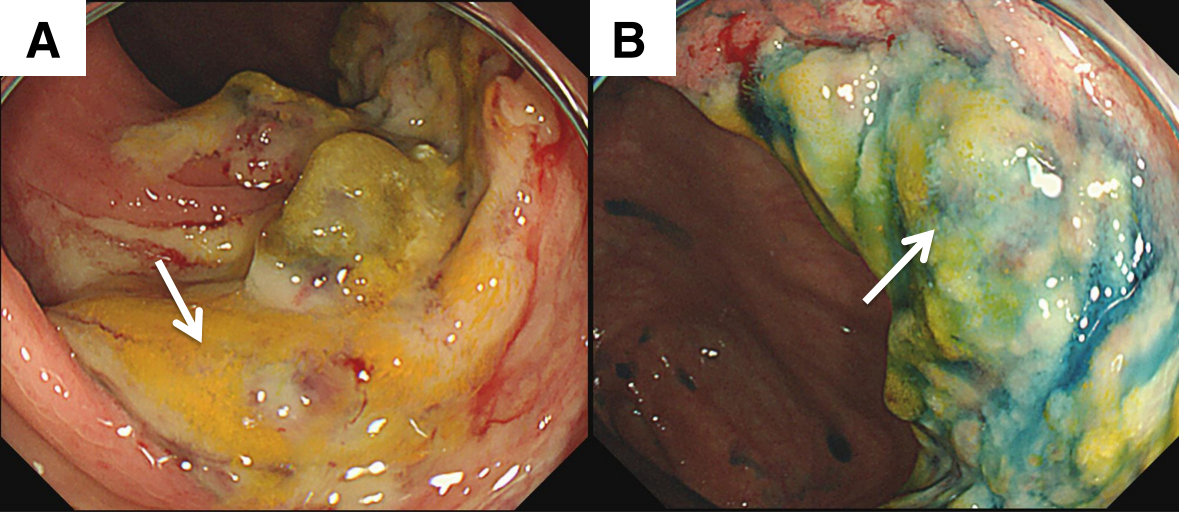
**a** A white coating could be seen in the vicinity of the Bauhin valve. **b** Localized distorted ulcers could be seen in the ascending colon. Mucosal necrosis was not found

**Fig. 2:**
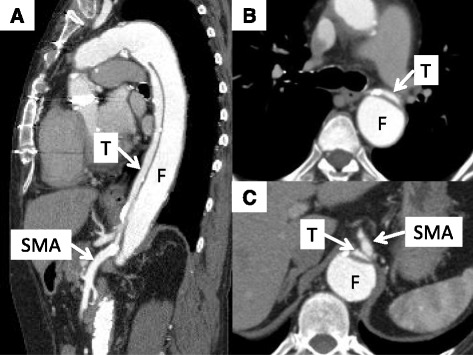
The true lumen (*T*) and superior mesenteric artery (*SMA*) were compressed by the expanded false lumen (*F*). **a** Sagittal slice, **b** axial slice at the level of bronchial bifurcation, and **c** axial slice at the level of the SMA. *T* true lumen, *F* false lumen

Her initial abdominal pain with ischemic colitis occurred 1 year ago, and colonoscopy was performed. By the diagnosis of the colonoscopy, we found a white coating in the vicinity of the Bauhin valve and localized distorted ulcers in the ascending colon. However, mucosal necrosis was not found (Fig. 1). Since her symptoms were slight and temporary, she remained on conservative treatments at that time. Her abdominal pain became worse; melena occurred often during the course of her conservative treatments, and these symptoms occurred when blood pressure became low under hemodialysis. Then, she was referred to our hospital for further evaluation of her recurrent ischemic colitis. Computed tomography (CT) scans showed chronic B-AD, which extended from the distal anastomotic part of the total arch graft to both common iliac arteries. The distal re-entry size was 26 mm. The expanded false lumen compressed the true lumen of most of the aorta, including the origin of the superior mesenteric artery (SMA) (Fig. 2).

**Fig. 3:**
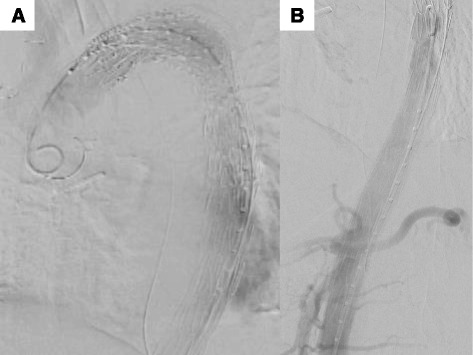
**a** The TX2 was deployed just distal to the left subclavian branch to seal the entry of the false lumen. **b** The TXD was deployed at the SMA to expand the true lumen and improve blood flow of the SMA

Based on these clinical findings, we considered that this recurrent ischemic colitis was a result of diminished blood flow to the SMA due to the compressed true lumen combined with hypotension due to hemodialysis. We then determined that therapeutic enlargement of the true lumen would improve blood flow to the SMA and reduce the risk of ischemic colitis. For treatment, we decided to perform TEVAR because open surgical repair can be risky for the patient considering the history of previous Bentall operation and comorbid ESRF. Because the patient’s quality of life was adversely affected by recurrent ischemic colitis, she agreed to undergo the procedure we suggested, and informed consent was obtained. The operation was performed under general anesthesia. An incision was made in the right groin to expose the right femoral artery. A 6Fr sheath introducer was inserted percutaneously from the left common femoral artery for aortography. A 8Fr sheath introducer was inserted from the right exposed common femoral artery, and we delivered a guidewire and catheter with contrast radiography to pass into the compressed true lumen. The proximal entry tear in the patient was covered by a Zenith TX2 stent graft (diameter, 32–28 mm; mean length, 200 mm; Cook Medical, Bloomington, IN), supplemented by a noncovered aortic stent (TXD; diameter, 36 mm; length, 164 mm; Cook Medical) around both renal arteries, the SMA, and the celiac artery (CA). The TX2 was deployed just distal to the left subclavian branch to seal the entry tear (Fig. 3a). The noncovered aortic stent (TXD) was deployed from above the CA to under both renal arteries to expand the true lumen and to improve blood flow of the CA and SMA (Fig. 3b). The operation time was 2 h and 33 min, and blood loss was 150 cc. The patient tolerated these procedures well and was transferred to the postoperative care unit in a hemodynamically stable condition. Her bowel condition improved just after the operation.

**Fig. 4:**
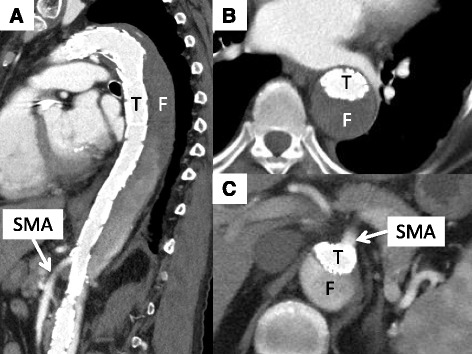
Blood flow of the true lumen and SMA was improved. The false lumen remained but tended to be thrombosed. **a** Sagittal slice, **b** axial slice at the level of bronchial bifurcation, and **c** axial slice at the level of the SMA. *T* true lumen, *F* false lumen

Seven days after the operation, CT showed improved blood flow in the true lumen, CA, and SMA, and the false lumen tended to be thrombosed (Fig. 4). She was discharged 10 days after the operation without any postoperative complications. She then underwent hemodialysis three times per week after the operation, but her abdominal pain following hypotension due to hemodialysis had completely disappeared. She remained well for 6 months after the operation.

### Discussion

We experienced successful TEVAR for complicated chronic B-AD. The patient had ischemic complications 8 years after the conservative treatments for chronic B-AD. This clinical course is rare because it took a long period of 8 years for ischemic colitis to appear after the occurrence of B-AD [7]. Furthermore, the occurrence of ischemic colitis on this patient was not easily recognized solely through the history of hemodialysis-dependent ESRF. Ischemic colitis is a well-known complication of ESRF [8], and abdominal pain after hemodialysis can then often occur because of hypovolemia. Preoperatively, in our patient, we observed that ischemic colitis under hemodialysis was coincidentally worsened by chronic B-AD. Interestingly, however, the abdominal ischemic symptoms disappeared completely after TEVAR. This clinical course suggested that the ischemic colitis was mainly caused by chronic B-AD and the relative hypovolemia following hemodialysis which had worsened the ischemic condition. Therefore, physicians should carefully evaluate the effect of stable chronic B-AD on ischemic colitis considering ischemic colitis may take a long period of time to appear after chronic B-AD and diagnosis of ischemic colitis is difficult.

With regard to treatment of B-AD, TEVAR is less invasive and an effective treatment compared with open surgery [9]. Fattori et al. demonstrated that operative mortality and complications were 33.9 and 40 % in open surgery compared with 10.6 and 20 % in TEVAR, respectively [10]. Hogendoorn et al. also reported that the 30-day mortality rate was 17.5 % in open surgery and 10.2 % in TEVAR [11]. In addition, TEVAR has better aorta-specific survival and delayed disease progression than conservative treatment [3]. Currently, TEVAR is a good treatment option for B-AD and is expected to be effective, even for complicated cases.

In the present case, the purpose of surgical treatment was to expand the true lumen and improve blood flow into the SMA. No aortic dilatation or rapid expansion was found in our case. We considered that our patient had many risks for surgical treatment because of her comorbidities. Therefore, we performed TEVAR with the TX2 in the proximal site to seal the entry tear and the TXD was deployed at the SMA to expand the true lumen and improve blood flow in the SMA. We did not expect that TEVAR above the CA could demonstrate the proper radial force to expand the true lumen below the CA because the dissection had occurred 8 years ago and the vessel wall could be highly sclerotic. In our case, organ ischemia was severe enough that an improvement of SMA blood flow was required. With regard to the distal site, the TXD which was composed of a noncovered aortic stent contributed to maintaining the opening of the origin of the SMA. At the same time, use of a bare metal stent allowed the remaining flow to the false lumen. In general, such endoleak is a risk for progression of aortic dilatation. TEVAR sometimes causes retrograde aortic dissection, which often requires immediate open surgical repair [12]. Therefore, covering the true lumen and extending into the thoracoabdominal aorta with a noncovered aortic stent to diminish retrograde endoleak could be important. In addition, flow to the artery of Adamkiewicz could contribute to prevent acute vertebral ischemia.

In our patient, TEVAR supplemented with a noncovered aortic stent was successful as the initial procedure. However, the long-term outcome is also important. The purpose of TEVAR for chronic B-AD includes not only improving prognosis but also preventing future surgical intervention of aortic dilatation [13]. Physicians need to be aware of other complications of TEVAR, such as stent-graft collapse, stent-graft migration, stent-graft torsion, aortoesophageal fistula, mobile thrombus within the stent-graft lumen, aneurysm formation, and late rupture. These complications warrant the importance of close and lifelong imaging follow-up of these patients [12]. Therefore, after TEVAR for B-AD, patients should be carefully followed up to identify any complications.

## Conclusions

We present a rare case of chronic B-AD with unique ischemic colitis, which worsened following hypotension due to hemodialysis. Our treatment with TEVAR supplemented with a noncovered aortic stent was successful. We believe that TEVAR is a good treatment option, even for complicated chronic B-AD, including especially chronic cases or cases causing organ ischemia. Further studies are required to validate the optimal indication and effectiveness of TEVAR for complicated chronic B-AD.

## Consent

Written informed consent was obtained from the patient for publication of this Case Report and accompanying images. A copy of the written consent is available for review by the Editor-in-Chief of this journal.

## Abbreviations

B-AD: Stanford type B aortic dissection
SMA: superior mesenteric artery
TEVAR: thoracic endovascular aortic repair
